# COVID‐19 disruption reveals mass‐tourism pressure on nearshore sea turtle distributions and access to optimal breeding habitat

**DOI:** 10.1111/eva.13277

**Published:** 2021-07-17

**Authors:** Gail Schofield, Liam C. D. Dickson, Lucy Westover, Antoine M. Dujon, Kostas A. Katselidis

**Affiliations:** ^1^ School of Biological and Chemical Sciences Queen Mary University of London London UK; ^2^ School of Biological Sciences University of Bristol Bristol UK; ^3^ Centre for Integrative Ecology School of Life and Environmental Sciences Deakin University Geelong Vic. Australia; ^4^ National Marine Park of Zakynthos Zakynthos Greece

**Keywords:** adaptive management, coastal squeeze, predator avoidance, reproductive fitness, risk allocation, science‐based evidence, tourism pressure, UAV

## Abstract

Quantifying the extent to which animals detect and respond to human presence allows us to identify pressure (disturbance) and inform conservation management objectively; however, obtaining baselines against which to compare human impact is hindered in areas where human activities are already well established. For example, Zakynthos Island (Greece, Mediterranean) receives around 850,000 visitors each summer, while supporting an important loggerhead sea turtle rookery (~300 individuals/season). The coronavirus (COVID‐19)‐driven absence of tourism in May–June 2020 provided an opportunity to evaluate the distribution dynamics of this population in the absence (2020) vs. presence (2018 and 2019) of visitors using programmed unmanned aerial system (UAS) surveys. Ambient sea temperature transitioned from suboptimal for breeding in May to optimal in late June, with turtle distribution appearing to shift from shallow (to benefit from waters 3–5°C above ambient) to deeper waters in 2018 and 2019, but not 2020. The 2020 data set demonstrated that increased tourism pressure, not temperature, drives turtles offshore. Specifically, >50% of turtles remained within 100 m of shore at densities of 25–50 visitors/km, even when sea temperature rose, with 2018 and 2019 data supporting this trend. Reduced access to warmer, nearshore waters by tourism could delay the onset of nesting and increase the length of the egg maturation period between nesting events (internesting interval) at this site. A coastal refuge zone could be delimited in May–June where touristic infrastructure is minimal, but also where turtles frequently aggregate. In conclusion, sea turtles appear capable of perceiving changes in the level of human pressure at fine spatial and temporal scales and adjusting their distribution accordingly.

## 1 INTRODUCTION

There is widespread evidence that many human activities disturb wildlife and damage associated habitats, including urbanization, agriculture and recreation in natural environments (Gaynor et al., 2018; Larson et al., 2016; Noon et al., 2012). Impacted animals often invest in risk avoidance behaviours, similar to predator avoidance, to evade contact with humans; however, such strategies carry potential costs of utilizing suboptimal resources or occupying suboptimal habitats, which negatively impacts their biology, reproductive success and, ultimately, survival (Frid & Dill, 2002; Gaynor et al., 2018; Valeix et al., 2012). Examples of evasive behaviours include changing habitat use or the timing of use, with many species exhibiting increased nocturnal activity (Cruz et al., 2018; Gaynor et al., 2018; Stillfried et al., 2015). It is very difficult to quantify such impacts directly, because baseline data on wildlife are often not available before human activities were introduced, limiting opportunities for science or evidence‐based conservation (Sutherland et al., 2004). However, unprecedented reduced human mobility during the COVID‐19 pandemic provided an unexpected opportunity to obtain baseline information on wildlife when human pressure is extremely low or, even, absent (Rutz et al., 2020). Such insights could improve our understanding of animal ecology and how to improve current conservation management once human pressure returns.

Obtaining information on human impact is particularly important for elusive and/or threatened wildlife (Noon et al., 2012); however, the technologies required to monitor their movement and behaviour at both individual and population levels are still emerging (Dujon et al., 2021; Hays et al., 2016, 2019), with marine animals being a classic example. Recreation and wildlife watching activities in coastal areas have been widely documented to negatively impact marine mammals, turtles and elasmobranches (e.g. Casale et al., 2020; Christiansen & Lusseau, 2014; Semeniuk et al., 2009); yet, such studies typically target individuals or small groups of animals in organized viewing settings, failing to capture potential responses at population levels (Christiansen & Lusseau, 2014; Larson et al., 2016; Papafitsoros et al., 2020). For example, while sea turtles are widely studied marine vertebrates globally (Hays & Hawkes, 2018), our understanding of their movement and behaviour is constrained by available technologies (Kays et al., 2015; Koh & Wich, 2012; Wilmers et al., 2015). The emergence of cheap, commercially available unmanned aerial systems (UASs) over the last five years has provided new opportunities to monitor marine vertebrates and invertebrates, including this group of seven sea turtle species, at population to regional levels in relation to their surrounding environment (for reviews, see Dujon & Schofield, 2019; Raoult et al., 2020).

Studies using various tracking and logging devices at sea turtle breeding areas (Rees et al., 2016) have repeatedly confirmed the fundamental importance of sea temperature (optimal sea surface temperature: typically 26–29°C; range 22–32°C) in regulating the onset of nesting activity (Almpanidou et al., 2016; Mazaris et al., 2008; Weishampel et al., 2010) and the interval between successive nesting events (e.g. Hamel et al., 2008; Hays et al., 2002; Hill et al., 2017; Weber et al., 2011), including associated energetic implications (Fossette et al., 2012). Loggerhead sea turtles (*Caretta caretta*) have the most temperate breeding distributions of all sea turtle species (Casale et al., 2018; Dodd, 1988); consequently, thermal selection is very important at sites on the limits of this range, such as the Greek island of Zakynthos (Mediterranean Sea). At this site, the window of opportunity for breeding is highly constrained (Margaritoulis, 2005; Schofield et al., 2013). Tracking studies at this site have shown that females access thermal hotspots close to shore that are up to 3–5°C warmer than the ambient sea temperature to accelerate egg development (Fossette et al., 2012; Schofield et al., 2009), but appear to shift to deeper waters as soon as ambient temperatures exceed 26°C. This behaviour was previously attributed to trade‐offs in accelerating egg development at higher temperatures with increased metabolic rate and the need to conserve energy (Fossette et al., 2012; Gangloff & Telemeco, 2018; Shine et al., 2005).

However, in addition to sea temperature, the number of people visiting Zakynthos also sharply rises over the same period during summer (Arianoutsou, 1988; Mazaris et al., 2009; Papafitsoros et al., 2020), potentially increasing pressure on turtles in the marine area through more encounters and greater noise pollution. Human pressure has been inferred through monitoring the responses of individual turtles to recreational activities at Zakynthos (Papafitsoros et al., 2020; Schofield et al., 2015) and other sites (Casale et al., 2020; Samuel et al., 2005). However, obtaining clear‐cut evidence that human activities alter the in‐water distribution of sea turtles at the population level is challenging (Rees et al., 2016; Schofield et al., 2020). At Zakynthos, this would require disentangling the effect of temperature and beach visitors (tourists), which became, unexpectedly, possible when the Greek Government banned all international flights and restricted nationwide movement until 1 July 2020 to combat COVID‐19. As highlighted by Rutz et al. (2020), this unprecedented ‘anthropause’ provides an opportunity to understand the linkages between human and animal behaviour that would otherwise not be possible. Thus, here, we evaluated the distribution dynamics of this sea turtle rookery when visitors were absent (2020) and present (2018 and 2019) using weekly programmed UAS surveys. We hypothesized that, if turtles shift offshore due to rising sea temperature, the number of visitors present would have no effect and vice versa. Our results are expected to provide baseline information on the linkages between human and animal behaviour, which could be used to facilitate sustainable, evidence‐based management.

## 2 METHODS

### 2.1 Study area and species

**FIGURE 1:**
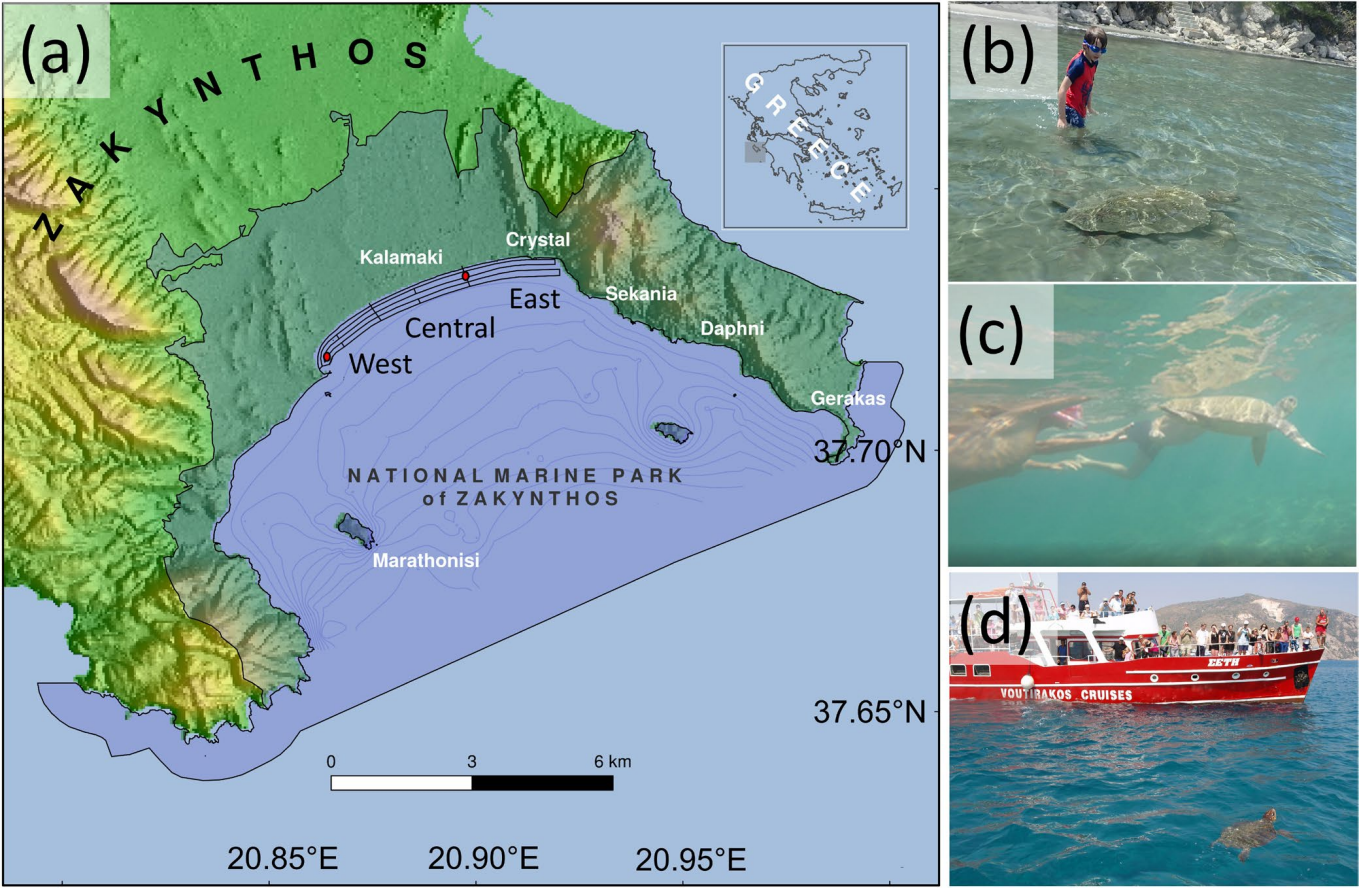
(a) Map showing the 6‐km UAS survey area in Laganas Bay (and the three sections, West, Central, East), within the National Marine Park of Zakynthos, Zakynthos Island, Greece. Black lines show the UAS paths; red dots show location of temperature loggers; and blue lines show 5‐m isobaths. Viewing turtles by (b) wading at 0.5 m seabed depth within 50 m of shore; (c) swimming at 1–2 m seabed depth within 50–200 m of shore; and (d) from a boat at 3–5 m seabed depth within 300–500 m of shore. Photograph credits: Gail Schofield

Zakynthos Island in Greece (Figure 1; 37°43′ N, 20°52′ E) supports an important breeding rookery for loggerhead sea turtles (*Caretta caretta*) in the Mediterranean Sea (Almpanidou et al., 2016; Casale et al., 2018; Casale & Margaritoulis, 2010), in parallel to being a major tourism destination, attracting over 850,000 visitors each summer (typically May to October) (Papafitsoros et al., 2020). The National Marine Park of Zakynthos (NMPZ) was established in 1999 (Figure 1a; Margaritoulis, 2005) to protect the breeding habitat of loggerhead sea turtles frequenting Laganas Bay, located on the southern part of the island. The protected area includes a terrestrial zone that protects the nesting habitat and a marine zone that protects female turtles between nesting events (internesting habitat). Female turtles frequent Laganas Bay from late April to early August, producing around 3–5 clutches each season (Schofield et al., 2013; Zbinden et al., 2007). Around 1244 clutches are produced on average each season (based on 23 years of data from 1984 to 2007; Casale & Margaritoulis, 2010), with an estimated 250 to 400 females and 100 males frequenting the area based on a 20‐year photo‐identification database and UAS records (Schofield et al., 2017, 2020). As such, this site has one of the highest numbers of nests per year for loggerhead sea turtles, and the highest nesting density, in the Mediterranean (Casale et al., 2018). Females typically return to breed every two years (range: 1–3 years), while males typically return to breed every year (range 1–2 years) based on tracking and photo‐identification data (Schofield et al., 2020). Males and females disperse to foraging grounds situated up to 1000 km from Zakynthos (Adriatic, Gulf of Gabes, Aegean and western Greece), with around one‐third of males remaining resident to the Ionian Sea (Schofield et al., 2020).

### 2.2 UAS surveys

Between 2018 and 2020, programmed UAS surveys were conducted at 1‐week intervals in May and June (*n* = 9 surveys/year) along a 6‐km nearshore stretch of water in Laganas Bay (Figure 1; based on Schofield et al., 2017). A DJI Phantom 3 Professional™ (Shenzhen, China; http://www.dji.com) was operated at 60 m altitude and at a speed of 12 m/s along preprogrammed line transects at 50 m, 150 m, 250 m and 350 m from shore (approximately representing 0.5 to 3.5 m seabed depth along the central line), providing a 100‐m‐wide field of view. All surveys were completed between 15:00 and 18:00, with this being the optimum period to detect turtles (Schofield et al., 2017). Intermittent checks to 800 m were conducted, to confirm the absence of turtle aggregations further offshore. We ran all transects in continuous flight mode and viewed the data during the processing stage only. Data were recorded in video format (3840 × 2160 pixels). All data were extracted using web‐based software (AirData UAV™). For additional details, see Schofield et al. (2017).

### 2.3 Data processing

All video footage was reviewed manually by two independent observers to extract information on sea turtles, people and vessels. The exact geographical coordinates (longitude, latitude) of each turtle, visitor and vessel were calculated from the position and timestamp on the computer images based on the GPS path of the drone recorded on‐board during flights. The shortest distance between turtles and people/vessels was also measured for each turtle, and whether the turtle was being viewed (termed encounter rate). A turtle was considered to be ‘viewed’ when two or more people/vessels surrounded the turtle in close proximity. This was validated on the ground during parallel photo‐identification surveys. Encounter rates were calculated as the percentage of turtles being viewed out of all turtles in each survey. The turtle and visitor data sets were evaluated on ESRI Arc GIS (ESRI, 2011; ArcGIS Desktop: Release 10.1, Environmental Systems Research Institute, Redlands, CA, USA) software.

### 2.4 Temperature measurements

Sea temperature was directly monitored using HOBO TidbiT v2 UTBI‐001Data Loggers (Onset Computer Corp.). Two loggers were placed in the marine area where turtles aggregate in the rookery (Figure 1a). Both in‐water loggers were placed 1 m below the sea surface at 3 m seabed depths to reflect temperatures typically experienced by a sea turtle at this site (Schofield et al., 2009). The loggers were set to record temperature at 15‐min intervals. The precision of the temperature loggers was 0.08°C of each other.

### 2.5 Calculation of nest numbers related to nearshore area use

Sea temperature regulates the number of days that turtles need to mature eggs before laying them, with >20 days required at 20°C, 12 days at 27–29°C (Hamel et al., 2008; Hays et al., 2002; Hill et al., 2017; Storch et al., 2005), and with upper limits of 30–32°C (Hill et al., 2017; Storch et al., 2005), above which metabolic rate (and hence energetic expenditure) and organ functioning are impacted (Fossette et al., 2012; Gangloff & Telemeco, 2018; Shine et al., 2005). Thus, turtles accessing optimal sea temperatures could lay more clutches than those in thermally suboptimal areas, assuming sufficient energetic reserves are accumulated during the foraging period (Broderick et al., 2001; Matsinos et al., 2008; Tucker, 2010).

On Zakynthos, the ambient sea temperature is suboptimal in May (17–22°C) and only reaches 26–28°C in late June (Schofield et al., 2009; present study), with the internesting period of tracked females generally declining from 22 days at the start of the nesting season to 13 days at the end of the nesting season (average 17 days across the period; Schofield et al., 2013; Schofield et al., 2015; based on GPS logging and tracking data of 62 females from pre‐ to postnesting period). However, we previously demonstrated that females occupying the 100‐m nearshore zone could access waters ≤5°C above ambient (Fossette et al., 2012; Schofield et al., 2009). This is reflected in our data, whereby some individuals consistently had internesting intervals <17 days throughout the nesting period, whereas others had intervals >20 days throughout the internesting period, indicating differences in thermal area use. These tracking data combined with 20 years of photo‐identification data also showed that females at this site produce at least 1 to 5 (mean: 3.4) clutches within a season, with 52% of females returning biennially and 21% annually, with the first and last nest of tracked individuals occurring in a mean 44‐day period (range: 30–68 days). This low clutch frequency relative to other, more tropical, sites globally, but noticeably high annual return rate (Casale & Ceriani, 2020; Esteban et al., 2017; Lee et al., 2018), indicates that energy stored by female turtles is not the only limiting factor and that reserves might not always be entirely depleted within a single nesting season (Matsinos et al., 2008; Troeng & Chaloupka, 2007; Wallace et al., 2007). Particularly in suboptimal nesting environments (like Zakynthos), the number of clutches is constrained by the thermal conditions of the site and the ability of females to optimize access to warm water (Matsinos et al., 2008; Schofield et al., 2009).

To calculate how the presence of turtles in the 100‐m zone (up to 5°C above ambient sea temperature) could theoretically impact reproductive output at the rookery level, we assigned different percentages of turtles in the rookery to this zone (to a maximum of 100%). We assumed a mean 325 females were present (Schofield et al., 2017, 2020) over 30‐day (lowest), 44‐day (mean) and 68‐day (maximum) nesting periods (Schofield et al., 2017), based on our published turtle tracking data sets (Schofield et al., 2013). We assumed that females in the 100‐m zone had access to optimal temperature (>27°C) throughout the nesting period, with internesting intervals of 13 days, while the remainder had partial (scenario 1) or no access (scenario 2) to ambient temperatures, reflecting the mean (17‐day) and longest (22‐day) internesting periods recorded for the tracked turtles, respectively. We then calculated the relative number of possible nests per season under each scenario.

### 2.6 Statistical analysis

We used the Anderson–Darling normality test to determine whether numerical variables (distance to shore of sea turtles and visitors) followed a normal distribution; the outputs showed that these parameters did not follow a normal distribution (A = 728.82, *p*‐value <0.01). Thus, we used the nonparametric Kruskal–Wallis test to investigate potential differences in turtle distance from the shore among the three years. We used the Dunn test for multiple comparisons of groups across years, with Benjamini–Hochberg adjustment, to evaluate significant differences. We used a multiple linear regression model to investigate potential associations of the percentage of sea turtles frequenting the area within 100 m of shore with visitor density, sea temperature and year. We tested for heteroscedasticity of the model residuals using a Breusch–Pagan test. The outputs showed that the residuals of the linear regression model showed no significant heteroscedasticity (BP = 0.82625, *p* > 0.05). We also tested the normality of residuals with a Shapiro–Wilk normality test, which showed that the residuals of the linear regression model follow a normal distribution (W = 0.9386, *p* > 0.05). The area within 100 m of shore was selected because it encompassed >90% visitors in all three years. We used R version 3.6.3 (R Core Team, 2018) for the statistical analysis and to create maps.

## 3 RESULTS

### 3.1 Temporal trends in sea turtles, visitors and sea temperature

**FIGURE 2:**
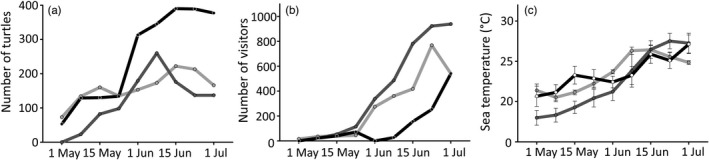
(a) Number of sea turtles and (b) number of visitors obtained from UAS video footage, and (c) mean weekly sea temperature (± standard deviation) obtained from in‐water temperature loggers during the May–June surveys of 2018 (light grey lines), 2019 (dark grey lines) and 2020 (black lines)

Overall, 3809 sea turtle locations and 7505 visitor locations (of which 15% were vessels with and without motors) were recorded over the three‐year period (8–9 surveys per year). Sea turtle numbers increased through May and peaked in early June for all three years (222, 260 and 390, respectively) and then noticeably dropped in 2018 and 2019 (Figure 2a). Visitor numbers were 2 to 3.5 times higher (May and June, respectively) in 2018 and 2019 (peak survey number 768 and 938, respectively) compared to 2020 (peak survey number 253 in June, rising to 540 on 1 July) (Figure 2b). Sea temperature increased from 17–22°C in early May to 25–28°C in late June in all three years (Figure 2c).

### 3.2 Spatial distribution of turtles and visitors

**FIGURE 3:**
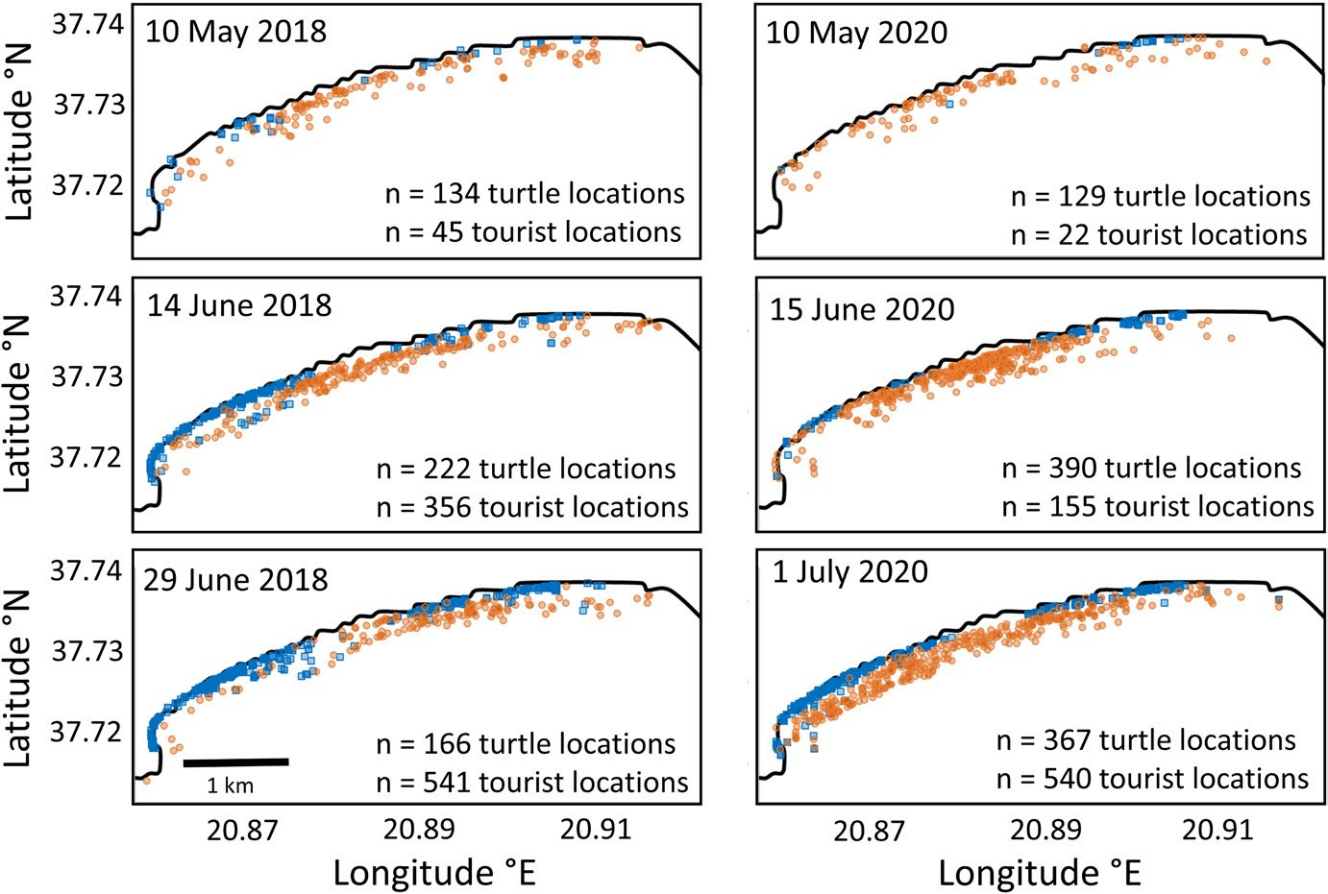
Example maps showing turtle (brown opaque circles) and visitor (blue opaque squares) distributions, along the 6‐km survey area and from 0 to 400 m offshore, across the survey period for 2018 and 2020. Note, 2019 followed similar trends as 2018, see Figure S1; days with similar environmental conditions were selected

**FIGURE 4:**
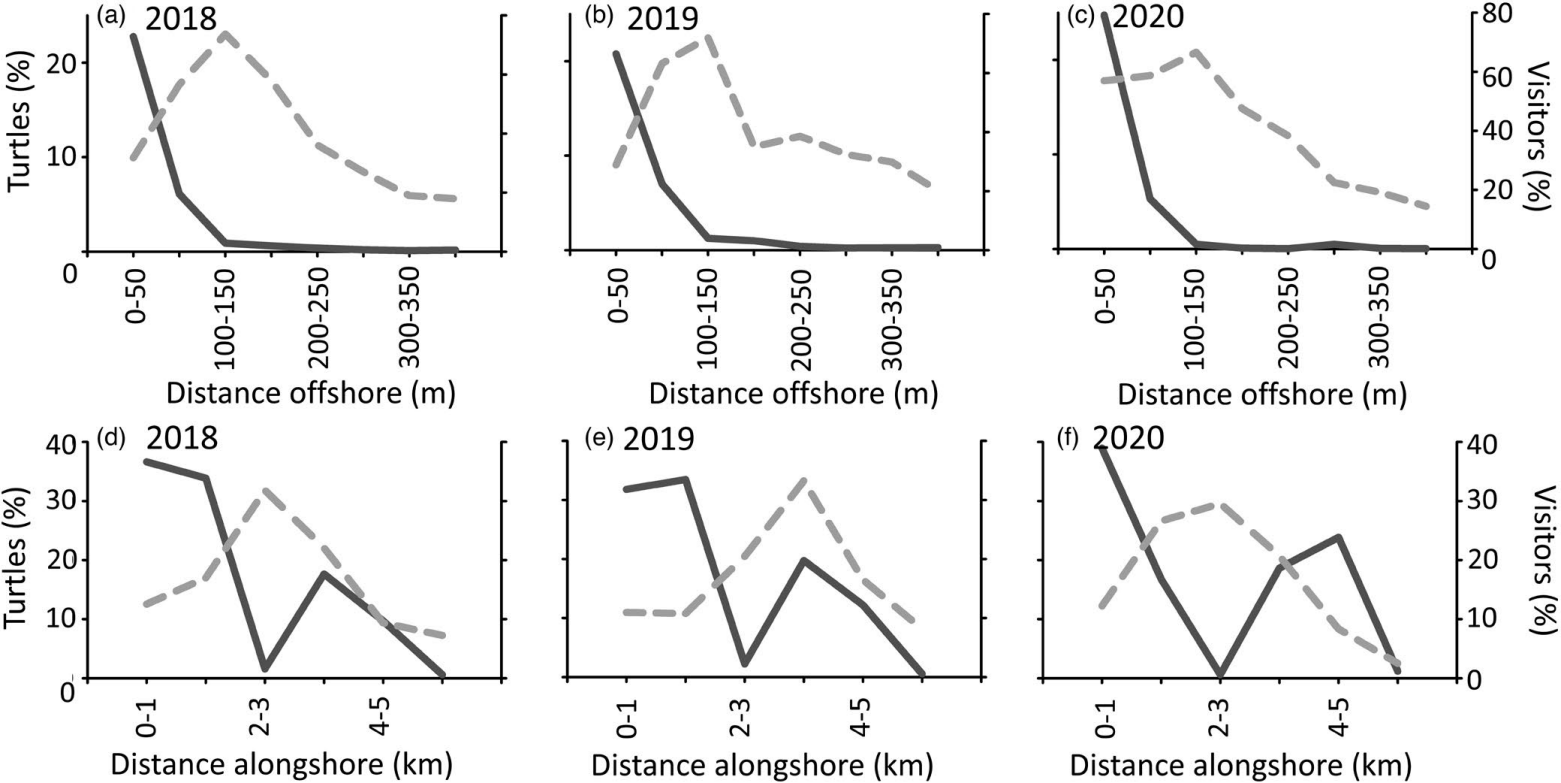
Percentage of turtles (dashed grey line) and visitors (solid grey lines, people and vessels combined, respectively) at 50 m groupings offshore (0–400 m) in (a) 2018, (b) 2019 and (c) 2020. Percentage of turtle (dashed grey line) and visitors (solid grey line) at 1‐km groupings alongshore from west to east in the survey area (20.86–21.90° longitude) in (d) 2018, (e) 2019 and (f) 2020. Note, vessels were combined with visitors due to their low numbers (max 15 within any 50 m bin)

For all three years, sea turtle numbers peaked at 100–150 m offshore and then declined to 400 m (Figures 3 and 4a–c). The Kruskal–Wallis test showed that the distance of sea turtles from shore significantly differed across the three years (Kruskal–Wallis chi‐squared = 25.239, *p* < 0.01). In both May and June 2020, 55% of turtles frequented the 0‐ to 100‐m zone, whereas this percentage was 37–43% in May dropping to 20–28% in June for 2018 and 2019. However, while there was no significant difference in sea turtle distance from shore between 2018 and 2019 (Dunn test, *Z* = −1.18, *p* > 0.05), both years were significantly different to 2020 (*Z* = 3.68, *p* < 0.01 and *Z* = 4.63, *p* < 0.01, respectively). In comparison, almost all visitors (wading, swimming) were concentrated in the 0‐ to 100‐m zone in all years (90% and 98% of visitors for 2018–2019 and 2020, respectively). However, while visitor density in the 0‐ to 100‐m zone was low in May for all three years (16, 21 and 4 visitors/km) and June for 2020 (33 visitors/km), it exceeded 100 visitors/km in June of 2018 and 2019. Vessels were almost entirely absent in 2020 (7 craft in total), while 164 and 242 were recorded in 2018 and 2019, which were widely spread across the 0–400 m zone (peaking at 200 m) (Figure 4a–c).

The alongshore distribution of visitors was similar in 2018 and 2019 (Figures 3 and 4d,e), with most visitors being concentrated along two distinct stretches (major tarmac road access points), separated by a 1‐km section with negligible visitor numbers in the middle (undeveloped sand‐dune system). This pattern existed in 2020 too, but with much lower numbers (Figures 3 and 4f). In comparison, alongshore turtle distribution differed across the three years. For instance, in 2018, turtles primarily frequented the 1‐km section where visitor numbers were low (8 ± 4 visitors/km for 2018 and 2019); however, in 2019, turtles primarily frequented an area where visitor numbers were high (around 20.89–20.90° longitude; 122 ± 40 visitors/km for 2018 and 2019).

Compared to 2020, turtle–visitor encounters were 3 to 5.5 times more likely in 2018 and 2019, respectively (*n* = 14 vs. 46 and 78, respectively), with 30–40% of encounters occurring within 100 m of shore and 80% within 200 m of shore. Encounters were higher where (1) visitor numbers were high and turtle numbers low (west section 2018 and 2019) and (2) both visitor numbers and turtles were high (i.e. around 20.89–20.90° longitude in 2019).

### 3.3 Sea turtles versus temperature and visitors

**FIGURE 5:**
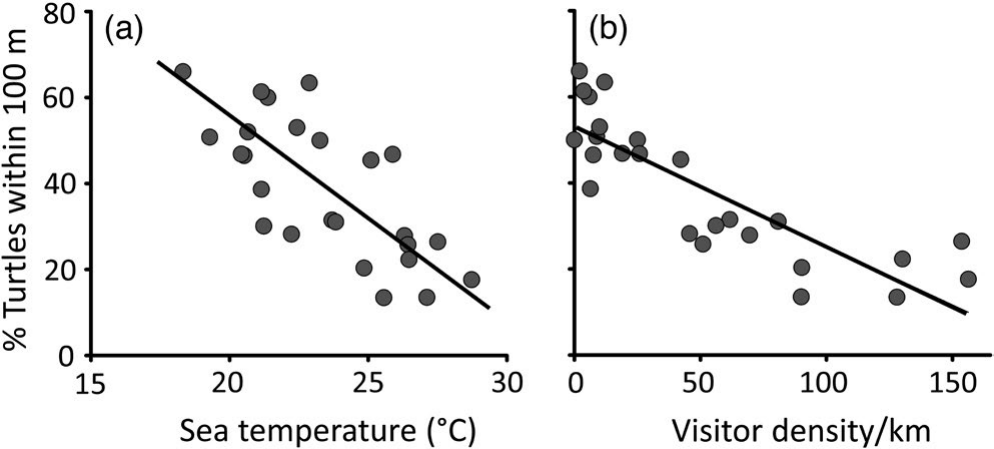
Percentage of sea turtles frequenting the area within 100 m of shore in the 2018–2020 surveys in relation to (a) ambient sea temperature (obtained from in‐water temperature loggers) (fitted linear regression line; *F*_2,24_ = 43, *r* ^2^ = 0.65, *p* < 0.01) and (b) visitor density (visitors/km) (fitted linear regression line; *F*_2,24_ = 55, *r* ^2^ = 0.84, *p* < 0.0001). Each point represents a survey date

The multiple linear regression model (*F*_3,22_ = 25.35, *p* < 0.01; *R* ^2^ = 0.7527) showed that the percentage of turtles within 100 m of shore was significantly positively associated with year (β‐coefficient = 4.62, *p* < 0.05) and significantly negatively associated with the density of visitors/km (β‐coefficient = −0.18, *p* < 0.05), but showed no association with sea temperature (β‐coefficient = −1.64, *p* > 0.05). However, when the two factors, ‘sea temperature’ and ‘visitor density’, were evaluated separately, sea temperature was significant (*F*_2,24_ = 43, *r* ^2^ = 0.65, *p* < 0.01; Figure 5a), but to a lesser extent than visitor density (*F*_2,24_ = 55, *r* ^2^ = 0.84, *p* < 0.0001; Figure 5b). The very strong association of sea turtle distance from shore with visitor density was revealed by the lower visitor density in 2020 compared to the other two years (Wilcoxon signed rank test *p* < 0.01). Based on Figure 5b, above a threshold of about 25–50 visitors/km, the percentage of turtles within 100 m of shore dropped from >50% to <50%.

### 3.4 Nest numbers related to area use

**FIGURE 6:**
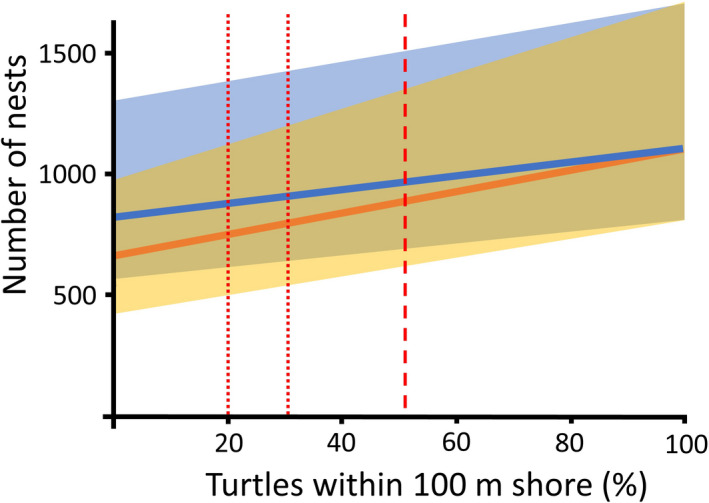
Calculated nest numbers on Zakynthos based on the percentage of turtles able to access the 100‐m nearshore zone with optimal temperatures (5°C above ambient) vs. ambient, with potentially 20–40% (250–500 nests on average) enhancement if 100% of turtles accessed the 100‐m zone throughout the breeding season and 8–15% (100–200 nests on average) enhancement based on comparative turtle distributions in the three studied years. Dotted red lines indicate the percentage of turtles using the 100‐m zone in 2018 and 2019, while the dashed red line indicates that for 2020. We assumed 325 turtles were present, with nesting periods of 30, 44 and 68 days (shaded areas, minimum–maximum; line, mean) for two internesting scenarios: (1) 13‐day intervals inside the zone and 17‐day intervals (mean) outside the zone (orange shading), and (2) 13‐day intervals inside the zone and 22‐day intervals (maximum recorded) outside the zone. Minimum, mean and maximum nesting period and internesting interval values were based on empirical data from 62 tracked females (Schofield et al., 2013), while female numbers were the average of predictions from photo‐identification and UAS surveys (Schofield et al., 2013, 2017, 2020)

Our calculations indicated that, theoretically, nest numbers at this site could be enhanced 20–40% (250–500 nests on average) if 100% of turtles accessed the 100‐m zone throughout the breeding season, based on the two scenarios (Figure 6). Section 3.3 showed that around 50% of turtles were documented in the 100‐m zone throughout 2020 vs. 20–30% in 2018 and 2019; thus, nest production could have theoretically been enhanced by 8–15% (100–200 nests on average) based on the two scenarios.

## 4 DISCUSSION

This study showed that sea turtles adjust their in‐water distribution to different levels of human pressure at fine spatial and temporal scales. While the alongshore distribution of turtles was driven by other factors, the proximity of turtles to shore was more strongly influenced by visitor numbers than sea temperature. It was only possible to obtain this insight on human–turtle linkage due to COVID‐19 disruption to the tourism industry in 2020 (Rutz et al., 2020). To avoid encountering humans, turtles moved into deeper waters, but potentially at the cost of losing access to optimal thermal conditions that accelerate egg maturation and shorten internesting intervals. Our data set showed that this shift in distribution occurs at a threshold of 25–50 visitors/km, which could be used as an evidence base to guide the conservation management of human pressure on sea turtles at other rookeries globally (Mazaris et al., 2009, 2014).

While there was a distinct 1‐km coastal stretch with very low human pressure (8 ± 4 visitors/km), and high visitor pressure on either side (Figures 3 and 4), our three‐year data set showed that turtles do not preferentially frequent this stretch as a ‘refuge’. We previously demonstrated that sea turtles change their alongshore distribution in response to wind‐driven transient thermal hotspots, that is they frequent the nearshore area at the down‐wind end (Schofield et al., 2009). Consequently, different combinations of daily prevailing winds each year likely led to the observed differences in predominant alongshore distribution of turtles, regardless of visitor pressure being consistently distributed. In contrast, the low visitor numbers in May and June of 2020 showed that tourism pressure, not sea temperature, regulates the offshore distribution of turtles. As documented in previous studies on human pressure (Frid & Dill, 2002; Stillfried et al., 2015; Valeix et al., 2012) and predator avoidance (e.g. Creel et al., 2005; Heithaus et al., 2012), turtles must make trade‐offs between accessing optimal resources and evading humans, supporting the risk allocation hypothesis (Lima & Bednekoff, 1999). This finding reinforces the importance of obtaining an evidence base of animal behaviour when human activities are absent, to identify such trade‐offs and quantify potential costs incurred (Papafitsoros et al., 2020; Rutz et al., 2020).

For instance, we previously assumed that turtles only moved into deeper waters as these waters became optimal for egg maturation (Schofield et al., 2009). However, here, we found that, when visitor pressure is low, turtles preferentially remained in warmer waters close to shore, even when sea temperature reaches the optimal temperatures elsewhere. Consequently, when visitor pressure is high, turtles might prematurely shift to deeper waters to evade humans. Because the window of opportunity for nesting on Zakynthos is highly constrained at the start of the nesting season (late May to late June) by suboptimal thermal conditions (Schofield et al., 2013, 2015), the onset of nesting and the duration of the internesting period (Hays et al., 2002; Hill et al., 2017; Mazaris et al., 2008) are strongly influenced by sea temperature. Furthermore, extending nesting later risks clutches failing to hatch when sand temperatures drop below the threshold for embryo development in September (Katselidis et al., 2012). Any females that lay more than three clutches at this site must access waters above ambient to accelerate egg maturation and shorten internesting intervals sufficiently (Fossette et al., 2012). During the early part of the season, sea temperatures warmer than ambient can only be accessed within 100 m of shore, with our calculations indicating that the extended use of this zone in 2020 (when tourism was absent) could have increased the total number of clutches by 8–15% (100–200 nests on average) compared to years with tourism. These calculated clutch frequencies reflected the those obtained from tracking studies of female loggerheads breeding on Zakynthos (Schofield et al., 2013; Zbinden et al., 2007). However, clutch frequency, and the number of eggs in each clutch, can be impacted by a number of biotic and abiotic factors independent of human pressure, including experience (e.g. knowledge acquired through life as detected in insects, fish and other reptiles; Finch, 2009; Kirkwood & Austad, 2000) and annual variation in reserves accumulated during foraging (Broderick et al., 2001; Matsinos et al., 2008; Tucker, 2010). Furthermore, any increase in ambient temperature at this site under climate change would likely enhance nest production, as temperatures closer to optimal would be reached earlier, with this phenomenon already being documented on Zakynthos and at other sites globally (Almpanidou et al., 2016; Mazaris et al., 2008; Patricio et al., 2021; Weishampel et al., 2010). Thus, caution should be taken when using nest counts to infer the actual numbers of female turtles frequenting thermally suboptimal breeding areas over protracted timeframes (Esteban et al., 2017; Mazaris et al., 2017; Pfaller et al., 2013).

Turtles moved further offshore when visitor pressure exceeded 25–50 visitors/km. This threshold provides a potentially useful evidence base for informing other areas supporting sea turtle rookeries, where human activities are currently in their infancy and informed conservation management decisions can be implemented (Hays et al., 2019; Larson et al., 2016; Mazaris et al., 2014). However, the National Marine Park of Zakynthos is situated within two major touristic coastal conurbations (Laganas and Kalamaki villages), reflecting the two core beach use zones by visitors (Figures 3 and 4); thus, restricting access to visitors during the daytime is not feasible and would not be supported by the local community (Togridou et al., 2006a). One option is to create a refuge zone for turtles along the central 1‐km zone, backed by sand dunes with limited road access, during May and June to limit disturbance, similar to that implemented for ground‐nesting birds on many beaches globally (e.g. Weston et al., 2012). However, our 3‐year data set showed that the alongshore distribution is primarily driven by bio‐physical parameters, not visitor pressure (Schofield et al., 2009). Thus, a refuge zone would only be partially effective across any given season (i.e. when conditions coincide with preferred turtle use); yet, it might also form an effective public awareness/education tool with associated economic benefits (Gregr et al., 2020; Papafitsoros et al., 2020; Togridou et al., 2006b). Studies using animal‐borne cameras have also demonstrated that sea turtles scope out potential nesting habitat during the daytime (Fuller et al., 2009). Thus, maintaining sections of coastal zones (beach and nearshore waters) with low human pressure might also promote nesting activity in these areas (Katselidis et al., 2013). In parallel, the strong overlap in human–turtle marine area use documented in 2019, and documented turtle–visitor encounter rates where visitor numbers are high, reaffirms the need to quantify the energetic impact of such encounters on female turtles and their reproductive output and hence fitness (Papafitsoros et al., 2020; Schofield et al., 2015; Senigaglia et al., 2016).

UAS technology made this study possible, allowing us to consistently capture turtle–visitor distribution dynamics at a representative scale over the course of three seasons. This study was also made possible by global COVID‐19 disruption, resulting in the Greek authorities restricting tourism until 1 July 2020. Consequently, obtaining more data under similar (low visitor pressure) conditions to 2020 is very unlikely. As stated by Rutz et al. (2020), this unprecedented ‘anthropause’ provided an opportunity to understand the linkages between human and animal behaviour that would otherwise not be possible, and is vital for shaping sustainable management, with the current study contributing quantitatively towards this. Field surveys were conducted at the optimal time of day for detecting turtles (i.e. afternoon; Schofield et al., 2009, 2017), which is also when the number of beach visitors tends to be highest (Arianoutsou, 1988; Togridou et al., 2006b). However, it should be kept in mind that each survey represents a snapshot (1 h) of a given day only. Our survey area was delineated based on remote tracking surveys and captures an estimated 65% of the breeding population (Schofield et al., 2017); however, it is possible that some turtles invest in other strategies outside the surveyed area or that the low tourist pressure in 2020 led to a greater number of turtles frequenting the survey area than usual.

In conclusion, visitor pressure appears to be the key factor driving the nearshore distribution of breeding female sea turtles, forcing a trade‐off between accessing warm water and evading visitors. Our study demonstrates the value of obtaining baseline information on animal behaviour in the absence of human activities and how the absence of access to such information hinders interpretation. Understanding how sea turtles perceive pressure (i.e. noise or encounters) and quantifying the energetic costs of moving offshore vs. remaining close to shore and being subject to human–turtle encounters is required in future studies, particularly to ascertain how these parameters impact clutch size and frequency. The fact that sea turtles can perceive changes in the level of human pressure, and adjust their distribution accordingly, has strong implications on how conservation management is implemented in coastal areas.

## CONFLICT OF INTEREST

The authors have no competing interests to declare.

## AUTHOR CONTRIBUTION

GS and KAK conceived the study and conducted the fieldwork. GS, LCDD and LW assimilated and processed the data. GS and KAK analysed the data. AD generated the code for the geographical locations. GS led the writing of the manuscript, with contributions from all authors.

## Supporting information

Fig S1

## Data Availability

The data that support the findings of this study are openly available in Dryad at https://doi.org/10.5061/dryad.n5tb2rbw6
