# Supplementary material for: COVID‐19 disruption reveals mass‐tourism pressure on nearshore sea turtle distributions and access to optimal breeding habitat
Source: Evol Appl. 2021 Jul 17;14(10):2516–26. doi: 10.1111/eva.13277 (PMC8444759; doi:10.1111/eva.13277)

**Supplement**

Supplementary Figure S1. Turtle and visitor distributions for similar dates to those shown in Figure 3 for 2018 and 2020.


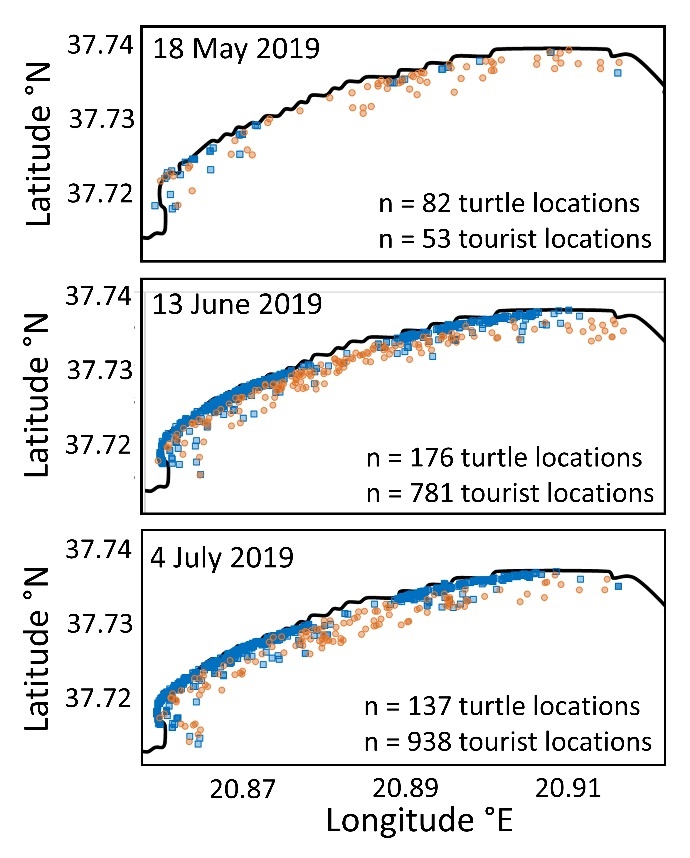

Supplement: Supplementary file 1 — Fig S1 [file EVA-14-2516-s001.docx]
